# Supplementary material for: A hybrid semiconducting organosilica-based O2 nanoeconomizer for on-demand synergistic photothermally boosted radiotherapy
Source: Nat Commun. 2021 Jan 22;12:523. doi: 10.1038/s41467-020-20860-3 (PMC7822893; doi:10.1038/s41467-020-20860-3)
Supplement: Supplementary file 1 — Supplementary Information [file 41467_2020_20860_MOESM1_ESM.pdf]

Supplementary Information

A hybrid semiconducting organosilica-based O_2 nanoeconomizer for on-demand synergistic photothermally-boosted radiotherapy

Tang et al.

Supplementary Figures

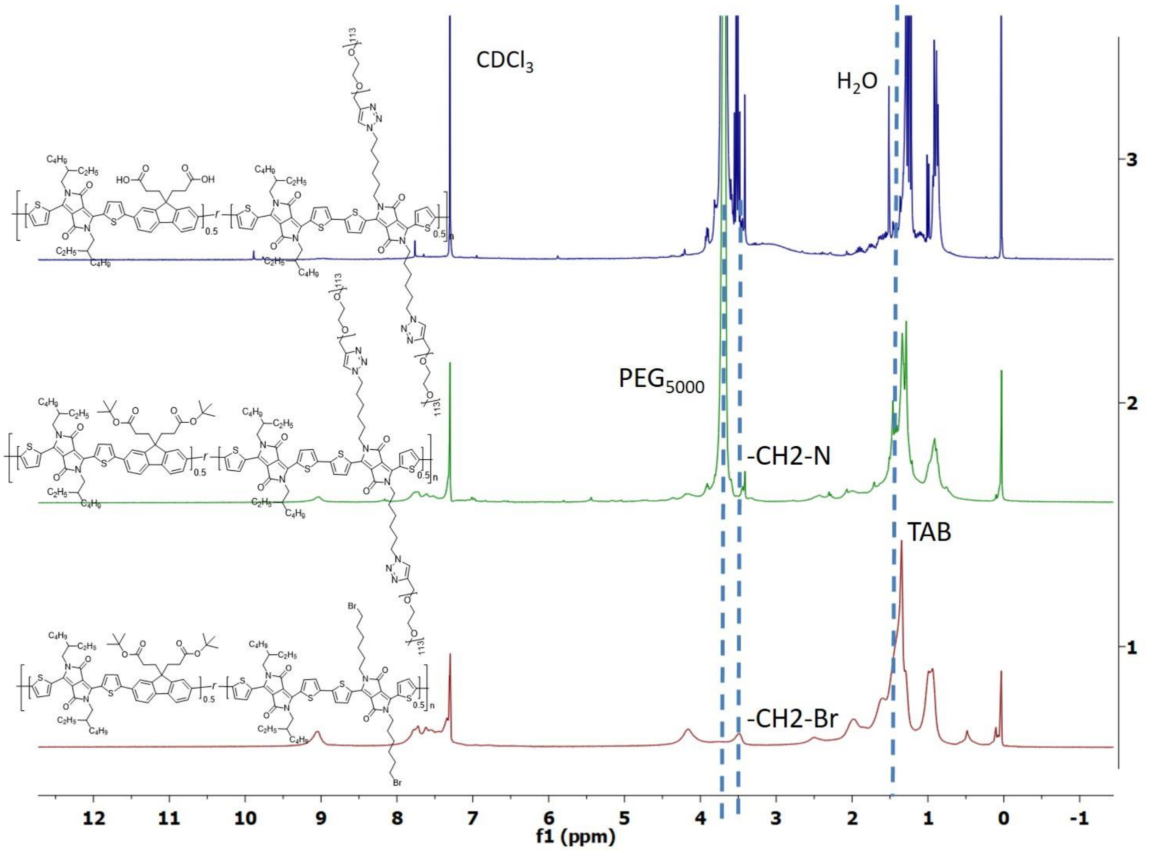

Supplementary Figure 1. ^{1}H-NMR spectra of SP-Br (bottom), SP-PEG$_{5000}$ (middle) and carboxyl group-based SPB (top) in $CDCl_3$.

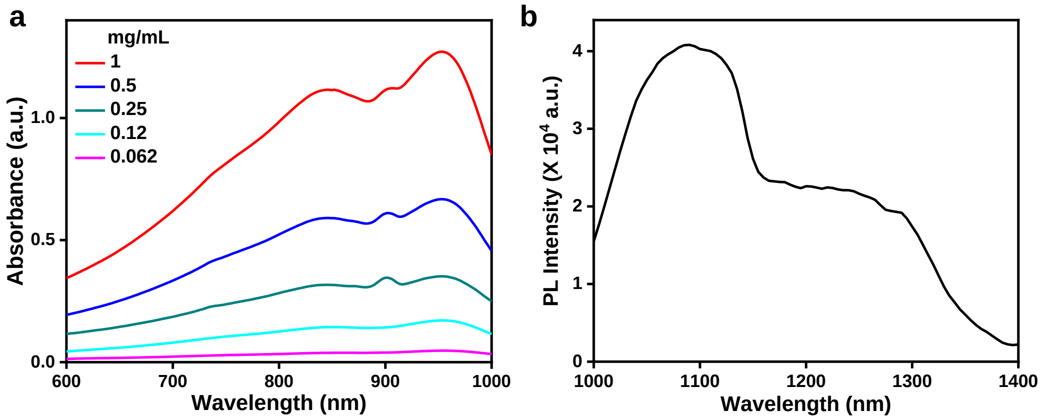

Supplementary Figure 2. (a) Absorbance spectra of SPBS at various concentrations. (b) NIR-II fluorescence spectrum of SPBS. Experiments were performed twice with similar

results.

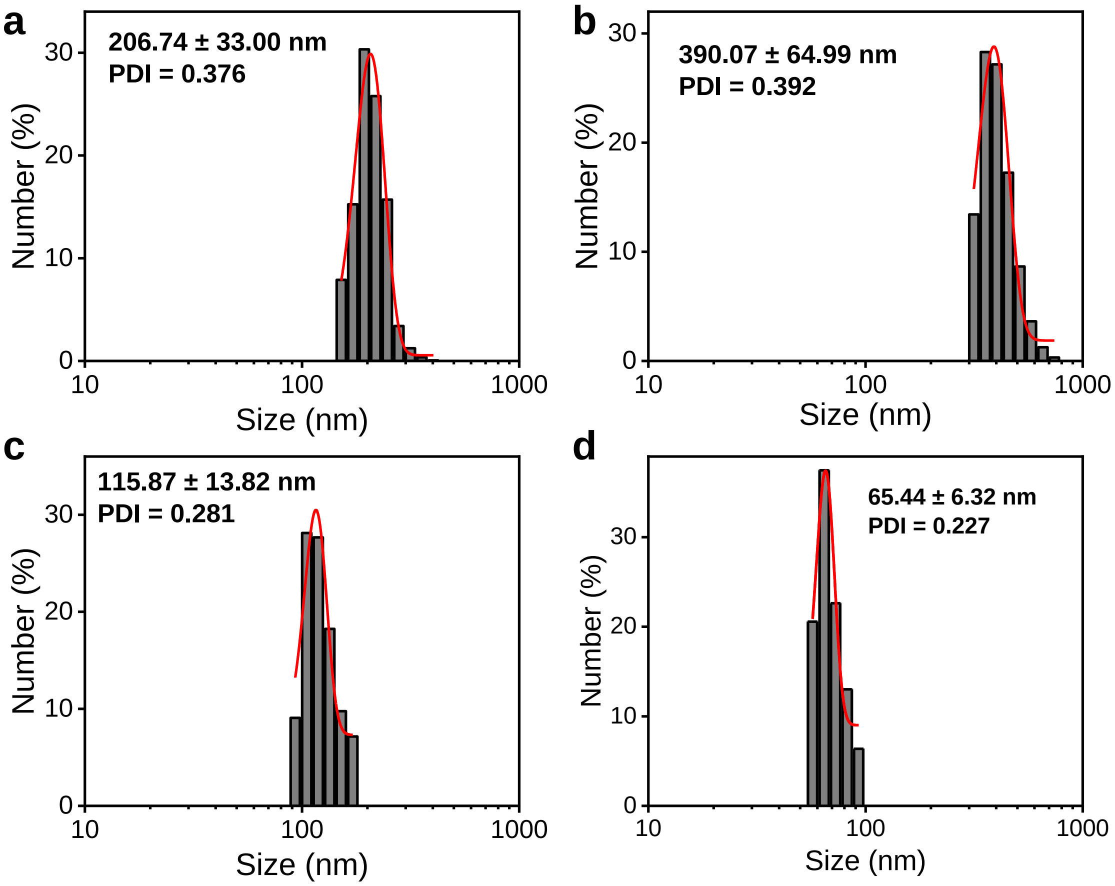

Supplementary Figure 3. Dynamic light scattering (DLS) size measurements of (a) SPB/FC-hybridized, (b) SPB/FC/(-Si-O-)-hybridized, (c) SPB/FC/thioether-hybridized, and (d) SPB/FC/phenylene-hybridized HPFON. Experiments were performed three times with similar results.

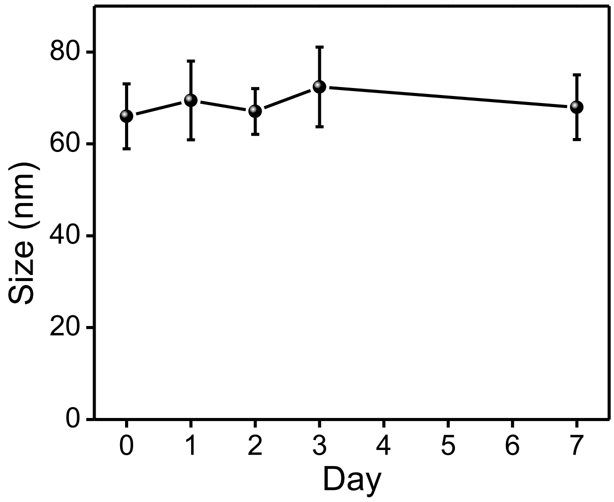

Supplementary Figure 4. DLS size measurements of semiconducting polymer brush/fluorocarbon/phenylene-hybridized HPFON over 1 week. n = 3 independent experiments. Data are presented as mean ± s.d.

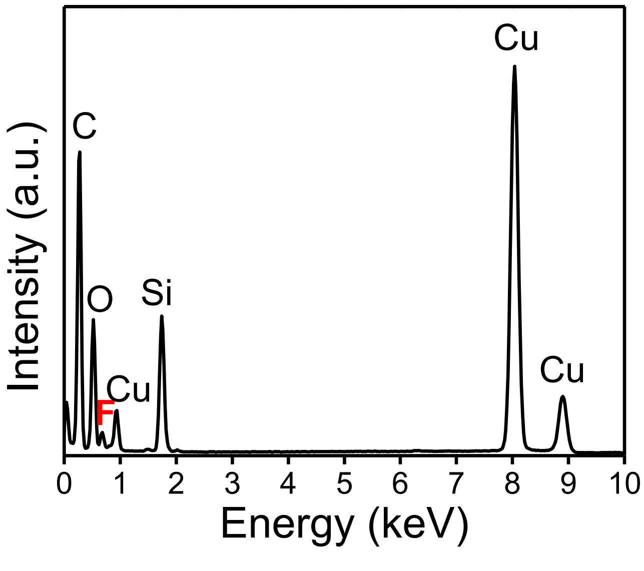

Supplementary Figure 5. Energy dispersive X-ray spectroscopy (EDS) spectrum of the semiconducting polymer brush/fluorocarbon/phenylene-hybridized HPFON. Experiments were performed three times with similar results.

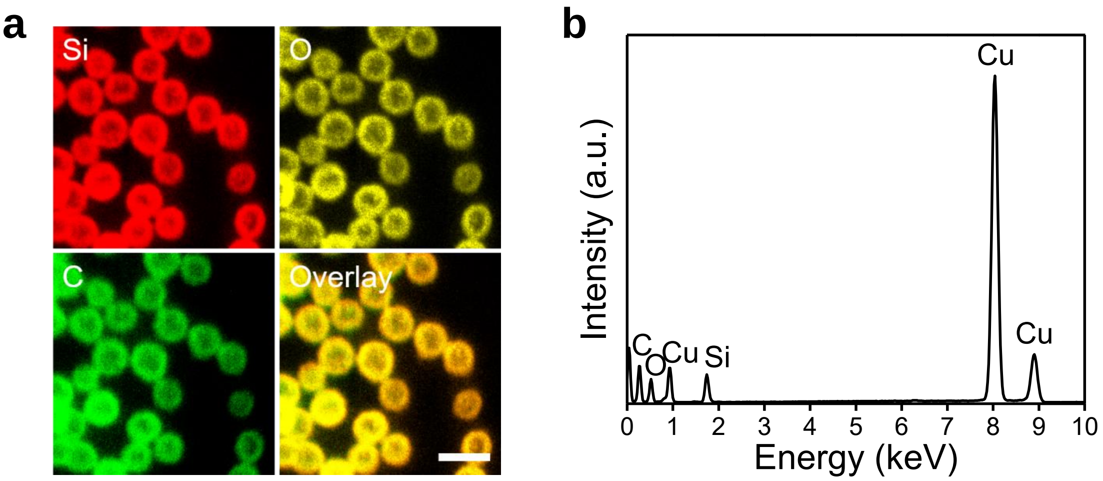

Supplementary Figure 6. (a) Elemental mapping analysis. Scale bar, 50 μm. (b) EDS spectrum of the semiconducting polymer brush/phenylene-hybridized HMON. Experiments were performed three times with similar results.

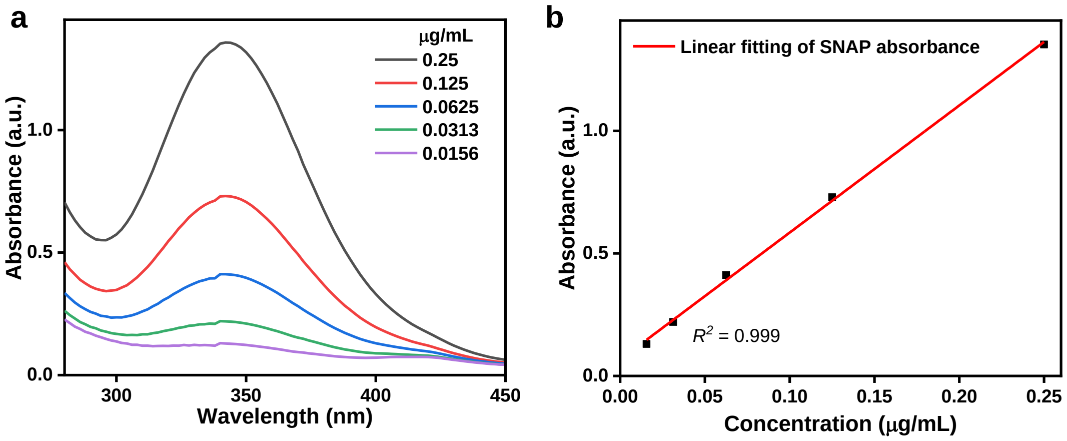

Supplementary Figure 7. (a) UV-Vis absorbance spectra of SNAP at various concentrations. (b) SNAP absorbance standard curve. Experiments were performed twice with similar results.

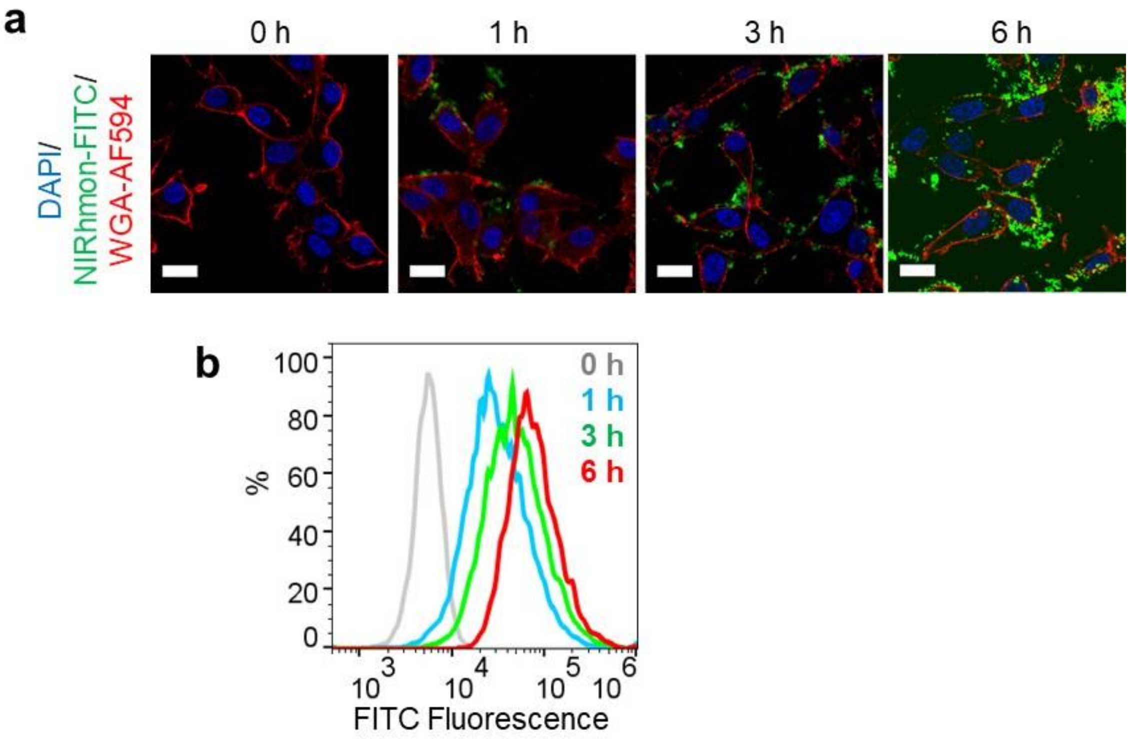

Supplementary Figure 8. (a) Confocal images of U87MG cells before and after incubation with FITC-labeled pHPFON for 1, 3, and 6 h. Green, FITC. Red, WGA-AF594. Blue, DAPI. Scale bar, 20 μm. (b) Flow cytometry analysis on the cellular uptake. Experiments were performed three times with similar results.

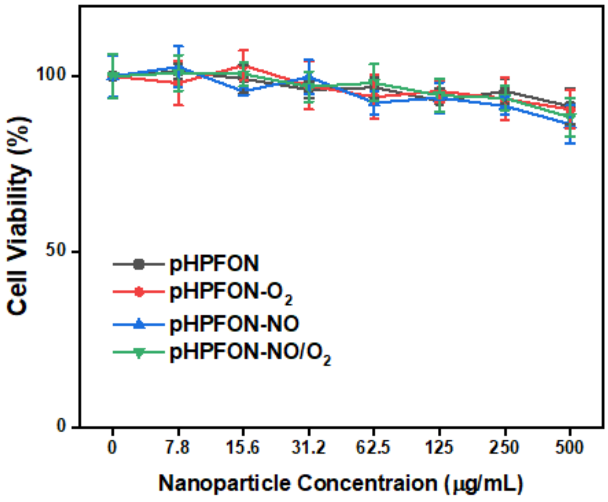

Supplementary Figure 9. MTT assays on U87MG cells after 24 h of incubation with pHPFON, pHPFON-O_2, pHPFON-NO, or pHPFON-NO/O_2. n = 5 biologically independent samples per group at each concentration. Data are presented as mean ± s.d.

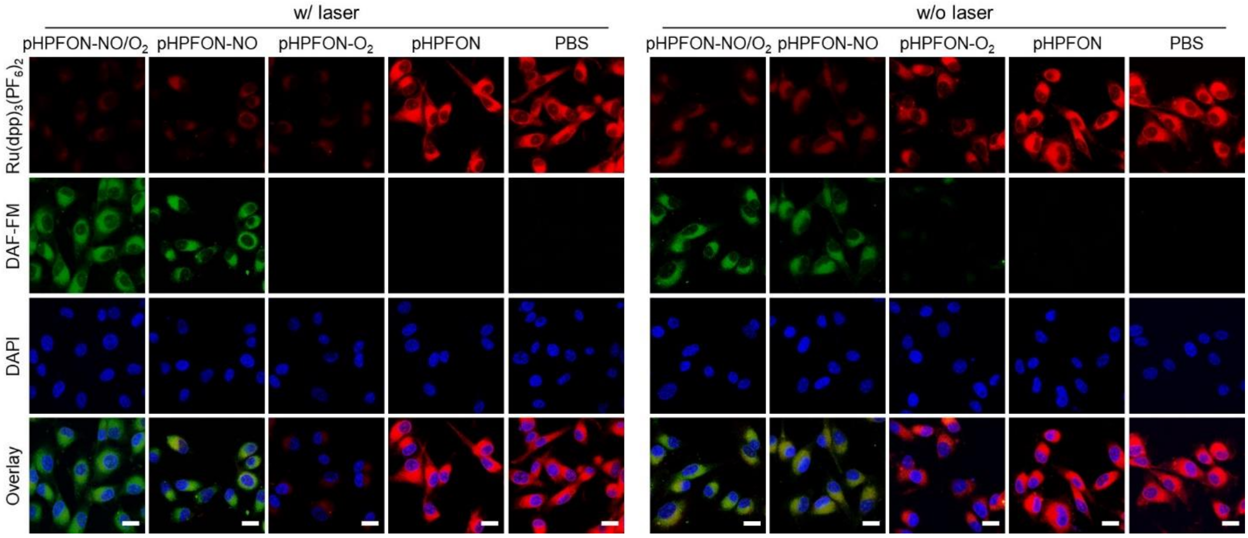

Supplementary Figure 10. Detection of NO and O_2 release in hypoxic U87MG cells treated with different pHPFON formulations for 24 h, with or without a following 808-nm irradiation. Green, DAF-FM (4-Amino-5-Methylamino-2',7'-Difluorofluorescein, NO indicator). Red, $[Ru(dpp)_3]Cl_2$ (hypoxia indicator). Blue, DAPI. Scale bar, 20 μm. Experiments were performed three times with similar results.

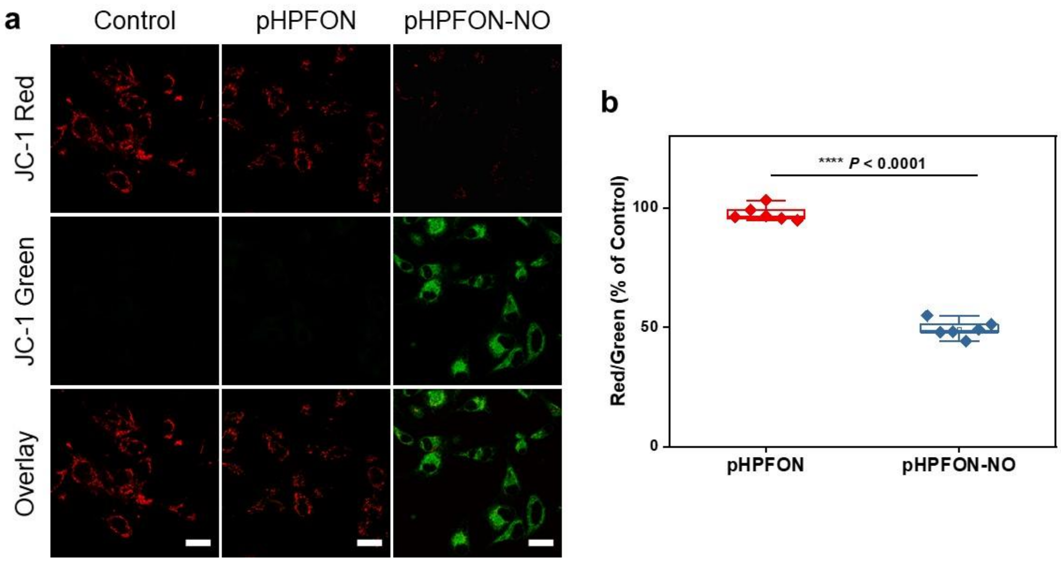

Supplementary Figure 11. JC-1 assays of hypoxic U87MG cells after 24 h of co-incubation with PBS, pHPFON, or pHPFON-NO. Green, JC-1 monomers, low mitochondrial membrane potential. Red, JC-1 aggregates, high mitochondrial membrane potential. Scale bar, 20 μm. Experiments were performed three times with similar results. (b) The ratio of red to green fluorescence in (a). n = 6 independent experiments. For the boxplots, the middle line is the median, the lower and upper hinges correspond to the first and third quartiles, and whiskers represent ± 1.5 inter-quartile range. Two-tailed Student's *t*-test. **** $P < 0.0001$.

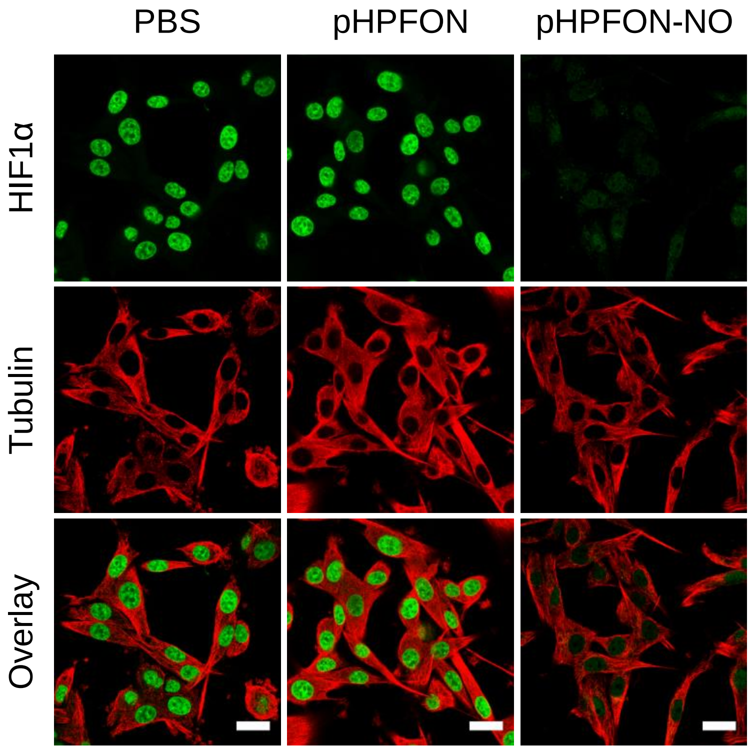

Supplementary Figure 12. Anti-HIF-1α staining on hypoxic U87MG cells after treating with PBS, pHPFON, or pHPFON-NO for 24 h. Green, HIF-1α. Red, tubulin. Scale bar, 20 μm. Experiments were performed three times with similar results.

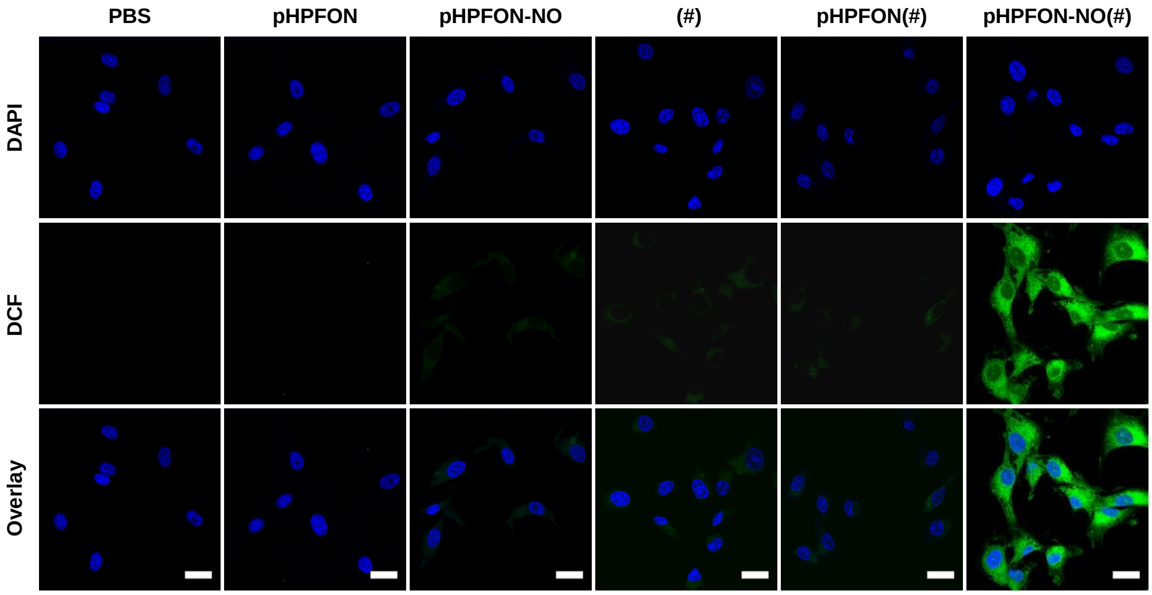

Supplementary Figure 13. Intracellular ROS generation in hypoxic U87MG cells after

treating with PBS, pHPFON, or pHPFON-NO for 24 h, with or without a subsequent X-ray irradiation at 4 Gy. Green, DCF. Blue, DAPI. (#) stands for 4-Gy X-ray irradiation. Scale bar, 20 μm. Experiments were performed three times with similar results.

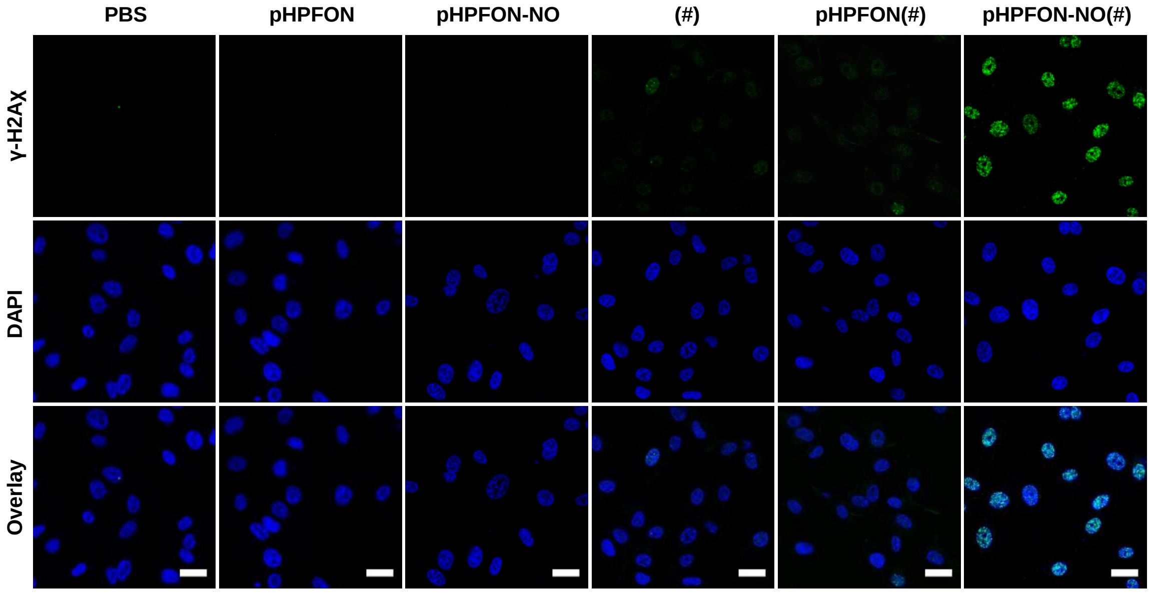

Supplementary Figure 14. DNA damage evaluation with anti-γ-H2Aχ staining in hypoxic U87MG cells after different treatments for 24 h, with or without a subsequent X-ray irradiation at 4 Gy. Green, γ-H2Aχ. Blue, DAPI. (#) stands for 4-Gy X-ray irradiation. Scale bar, 20 μm. Experiments were performed three times with similar results.

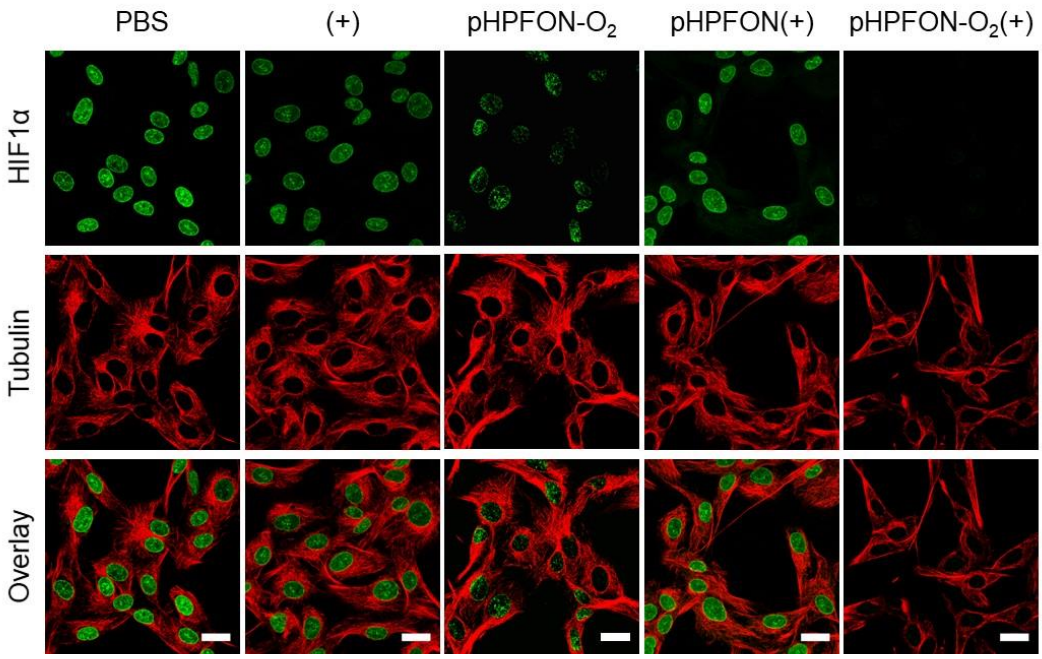

Supplementary Figure 15. Anti-HIF-1α staining of hypoxic U87MG cells treated with pHPFON-O_2 for 24 h and a subsequent laser irradiation (808 nm, 1 W/cm^2, 3 min). In controls, cells were treated with pHPFON + laser, pHPFON-O_2, + laser, or PBS. Green, HIF-1α. Red, tubulin. (+) stands for 808-nm laser irradiation at 1 W/cm^2 for 3 min. Scale bar: 20 μm. Experiments were performed three times with similar results.

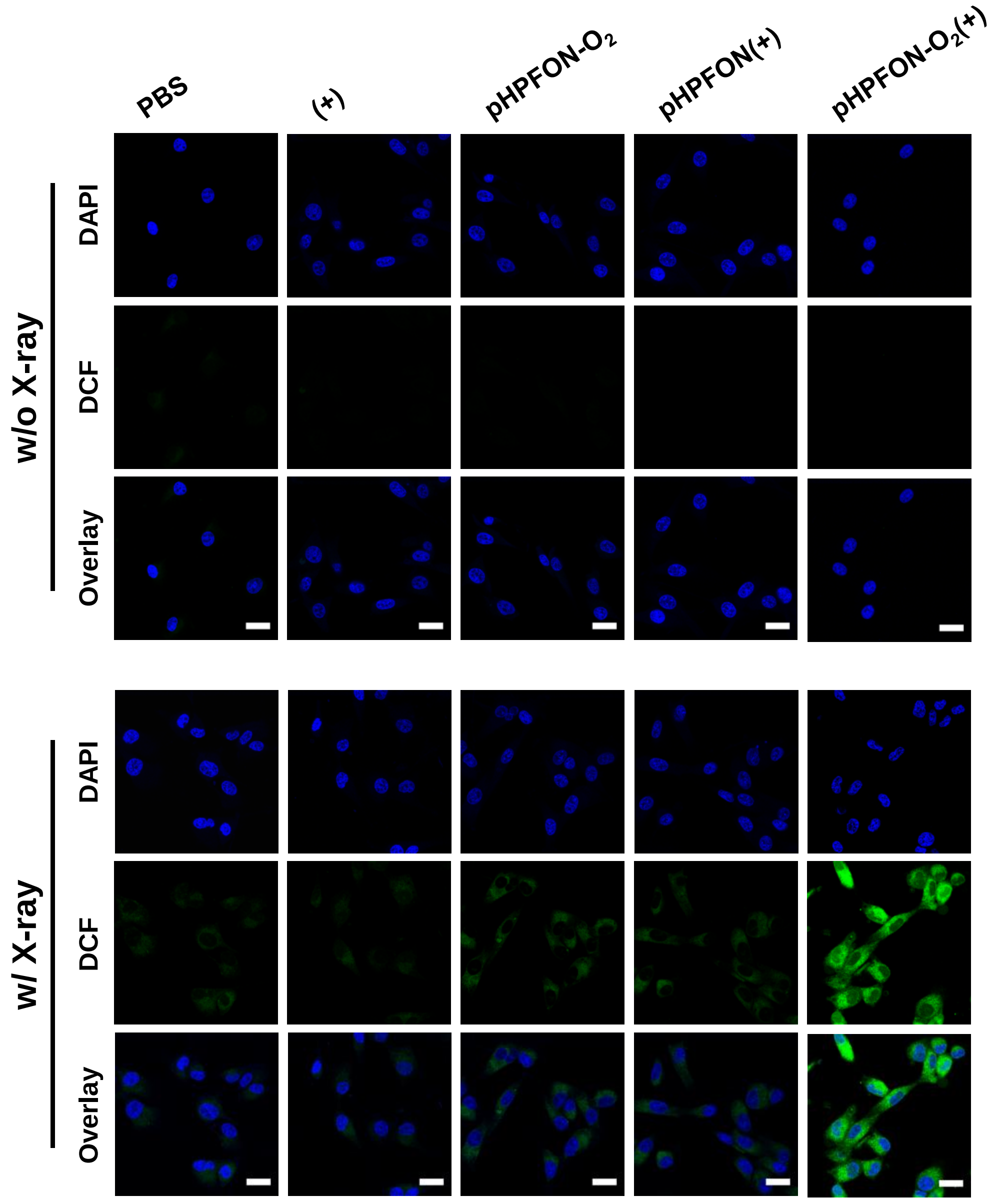

Supplementary Figure 16. ROS generation in hypoxic U87MG cells after receiving different treatments (pHPFON-O_2 + laser, pHPFON + laser, pHPFON-O_2, + laser, or PBS) with or without a following 4-Gy X-ray irradiation. Green, DCF (2',7'-dichlorofluorescein). Blue, DAPI. (+) stands for 808-nm laser irradiation at 1 W/cm^2 for 3 min, which applied after 24 h of nanoparticle incubation. Scale bar: 20 μm. Experiments were performed three times with similar results.

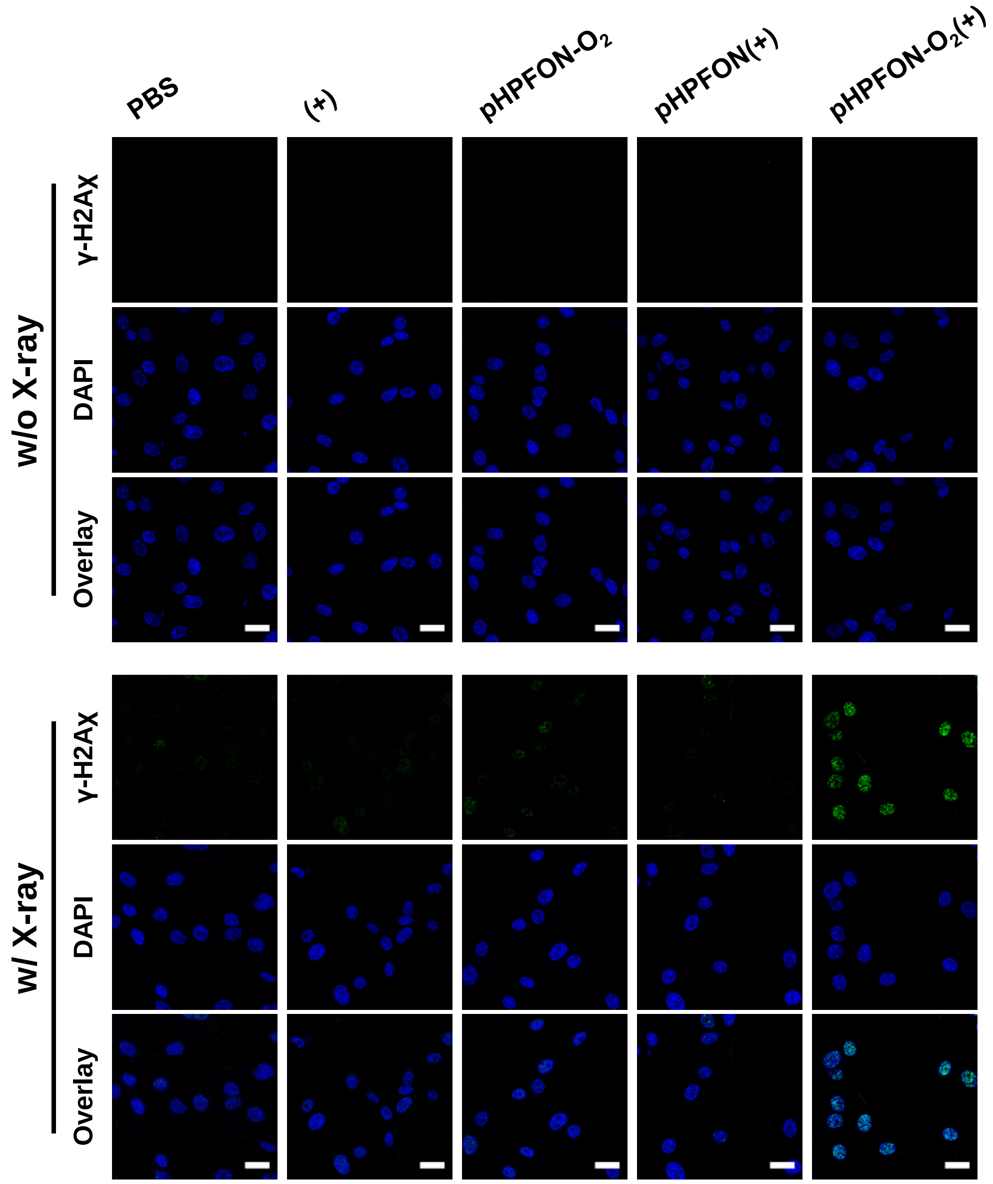

Supplementary Figure 17. DNA damage evaluation with anti-γ-H2Aχ staining in hypoxic U87MG cells after the treatments in Supplementary Figure 16. Green, γ-H2Aχ. Blue, DAPI. (+) stands for 808-nm laser irradiation at 1 W/cm^2 for 3 min, which applied after 24 h of incubation with nanoparticles. An X-ray irradiation was imposed at 4Gy after the laser irradiation, if applicable. Scale bar: 20 μm. Experiments were performed three times with similar results.

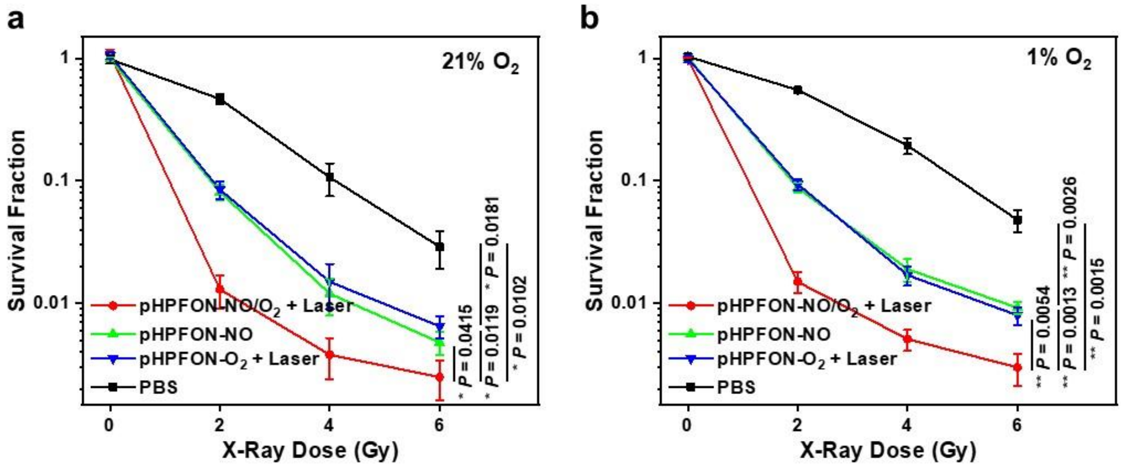

Supplementary Figure 18. Survival fraction determined by colony formation assays in both (a) normoxic and (b) hypoxic U87MG cells after different treatments. Laser was applied after 24 h of nanoparticle incubation at a dosage of 1 W/cm^2 for 3 min. X-ray was applied after the laser irradiation (if applicable) at various doses (0, 2, 4, and 6 Gy). n = 3 biologically independent samples per group. Data are presented as mean ± s.d. Two-tailed Student's *t*-test. * $P < 0.05$. ** $P < 0.01$.

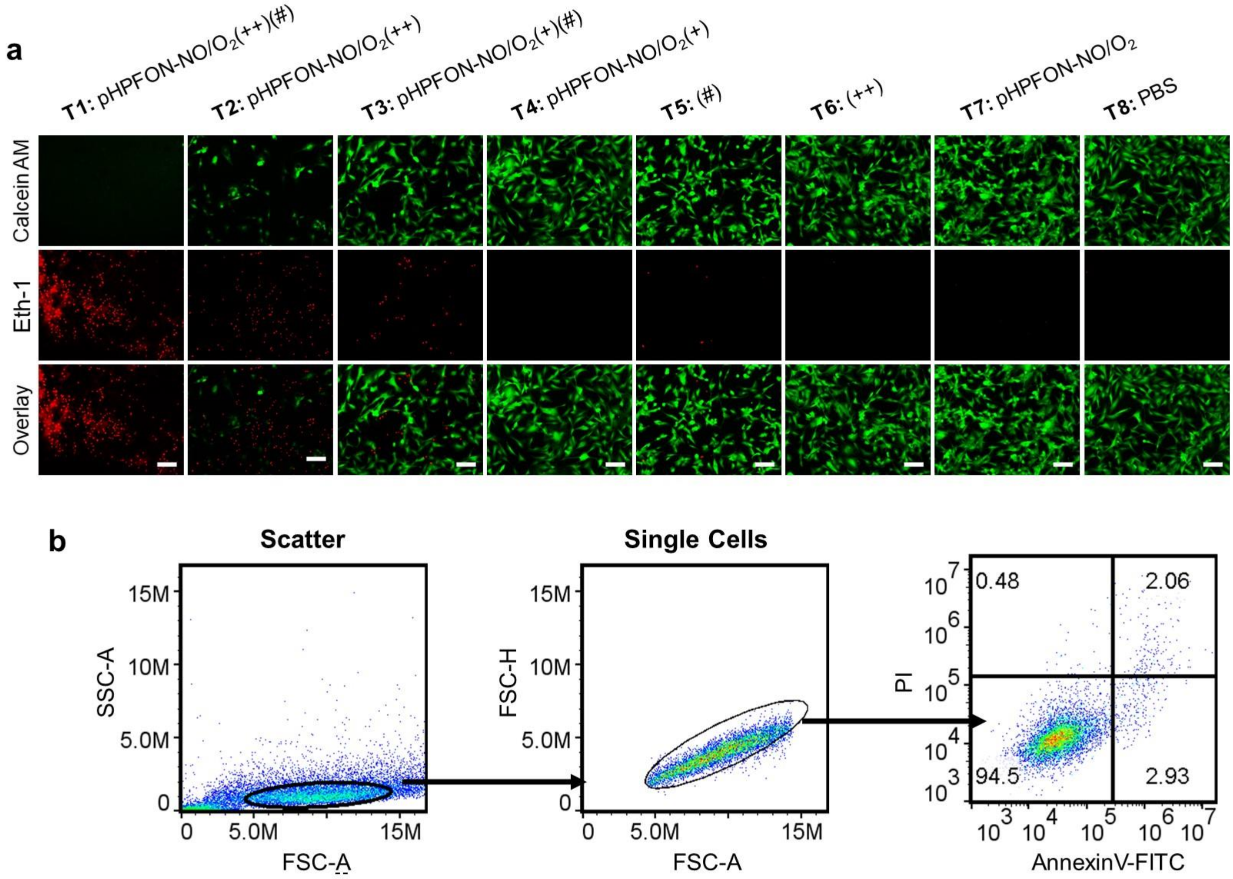

Supplementary Figure 19. (a) Live and dead assays on cells after different treatments. T1, pHPFON-NO/O_2(++)(#); T2, pHPFON-NO/O_2(++); T3, pHPFON-NO/O_2(+)(#); T4, pHPFON-NO/O_2(+); T5, (#); T6, (++); T7, pHPFON-NO/O_2; and T8, PBS. (++) indicates 808-nm laser irradiation at 1 W/cm^2 for 5 min. (+) indicates 808-nm laser irradiation at 1 W/cm^2 for 3 min. (#) indicates 4-Gy X-ray irradiation. Laser was applied after 24 h of incubation with nanoparticles and X-ray was applied after the laser irradiation, if applicable. Green, Calcein AM, live cells. Red, Eth-1 (ethidium homodimer-1), dead cells. Scale bar, 100 μm. Experiments were performed three times with similar results. (b) Representative flow cytometry gating strategies for FITC Annexin V-FITC/propidium iodide (PI) analysis in Figure 5h. Repeated for twice in independent experiments with similar results.

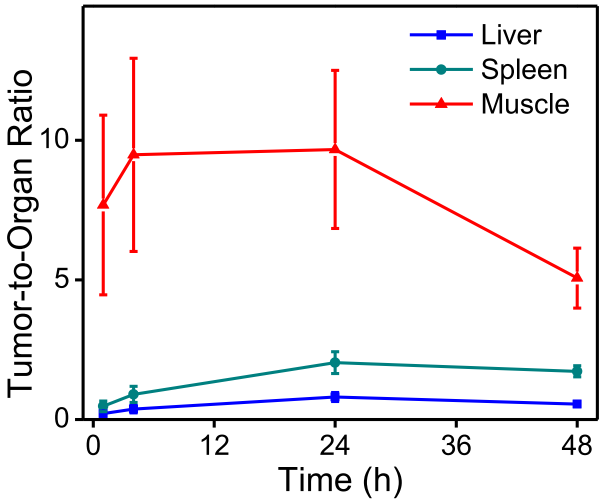

Supplementary Figure 20. PET quantification on tumor-to-organ ratio based on Figure 6a. n = 3 biologically independent animals. Data are presented as mean ± s.d.

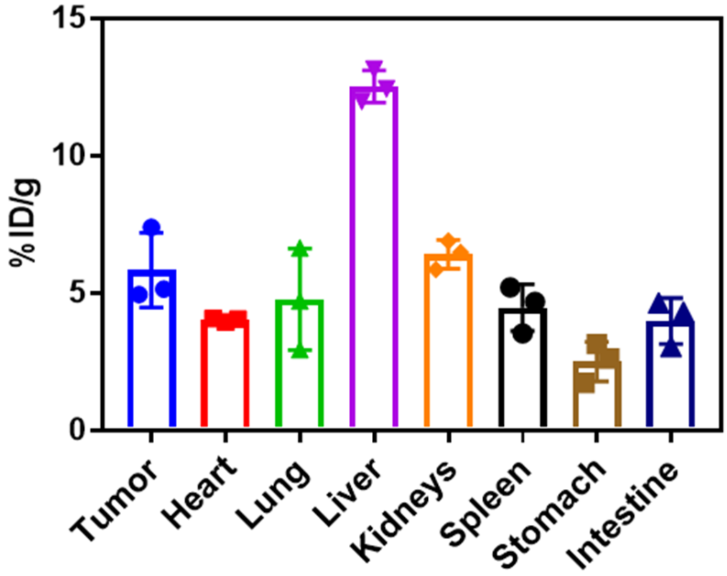

Supplementary Figure 21. Bio-distribution of the ^{64}Cu-labeled pHPFON-NO/O_2 at 48 h p.i. n = 3 biologically independent animals. Data are presented as mean ± s.d.

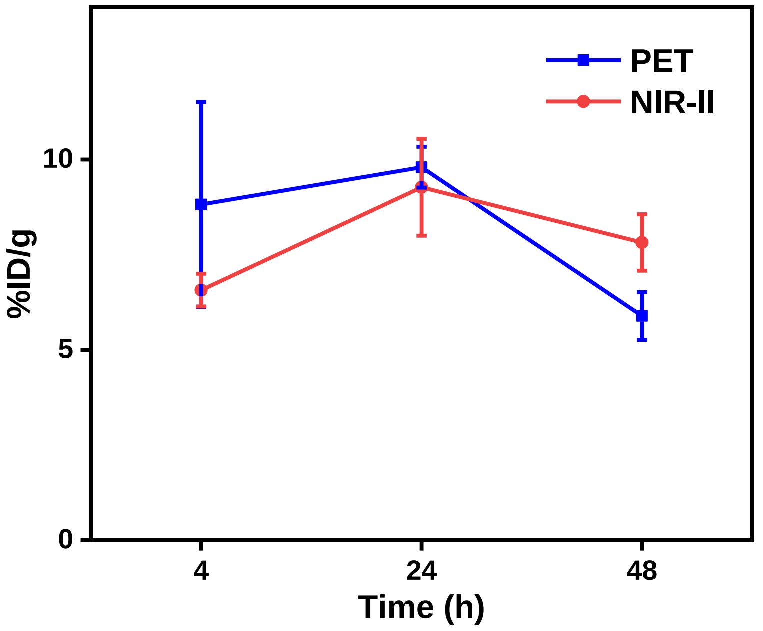

Supplementary Figure 22. Comparison of tumor accumulation of pHPFON-NO/O_2 under PET and NIR-II imaging based on Fig. 6 a and c. n = 3 biologically independent animals. Data are presented as mean ± s.d.

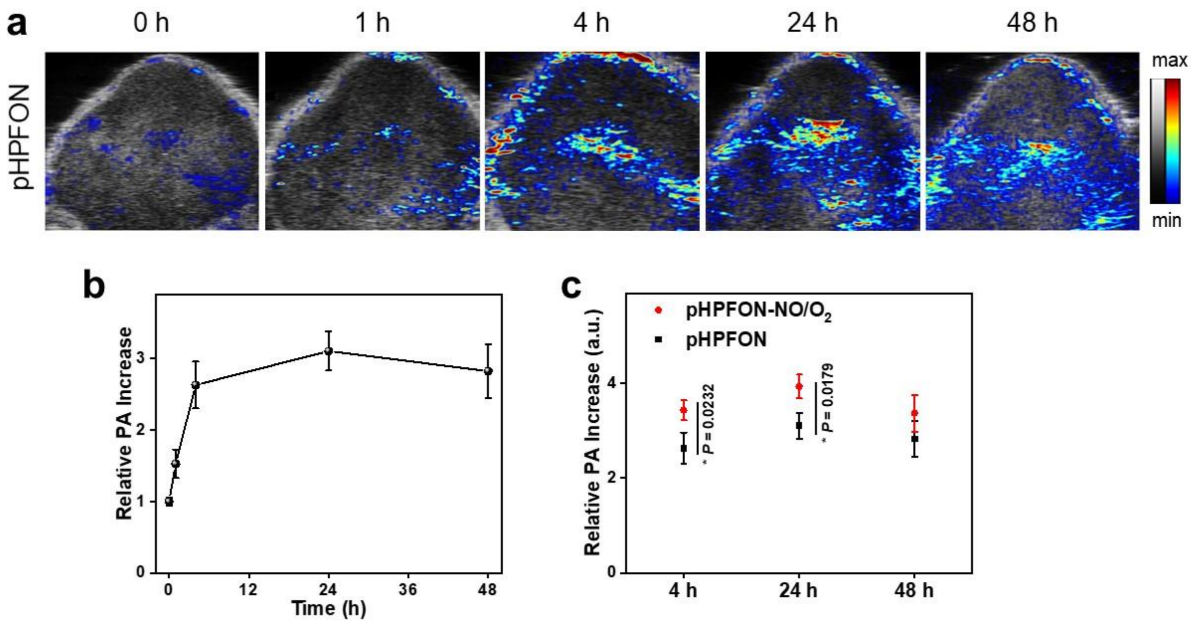

Supplementary Figure 23. (a) Representative PA images of U87MG tumors taken before

and at different time points after intravenous injection of pHPFON. (b) Relative PA signals in the tumor areas based on (a). (c) Comparison of relative PA signal increase at 4, 24, and 48 h p.i. between pHPFON-NO/O_2 and pHPFON. n = 3 biologically independent animals. Data are presented as mean ± s.d. Two-tailed Student's *t*-test. * $P < 0.05$.

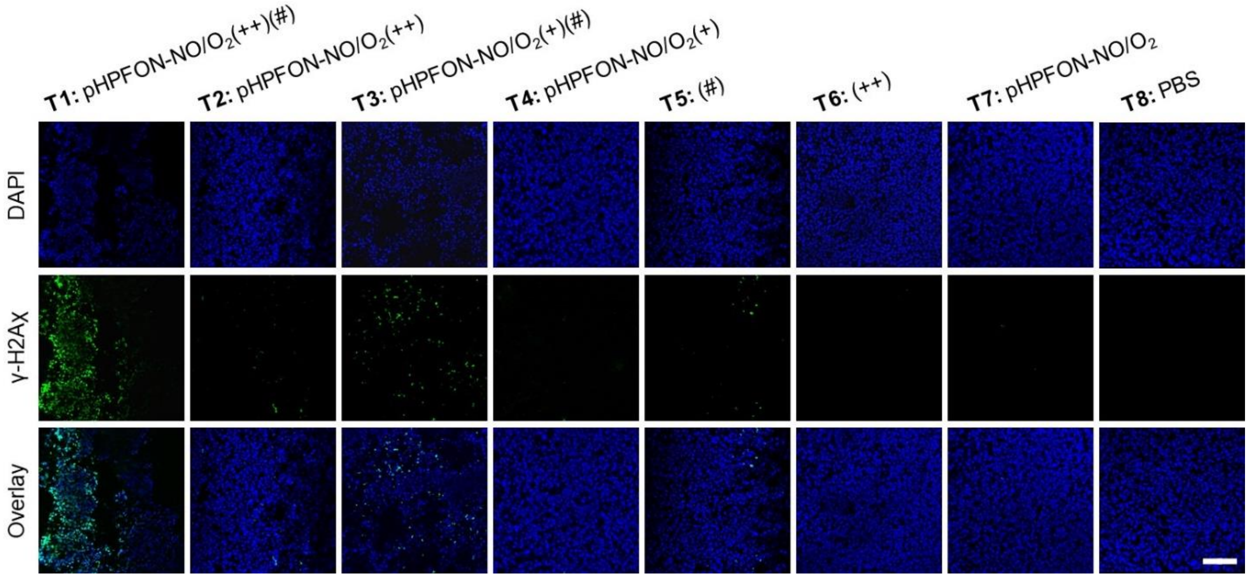

Supplementary Figure 24. Anti-γ-H2Aχ staining on tumor samples acquired at 30 min after different treatments in Fig. 7g. Green, γ-H2Aχ. Blue, DAPI. Scale bar, 100 μm. Experiments were performed three times with similar results.

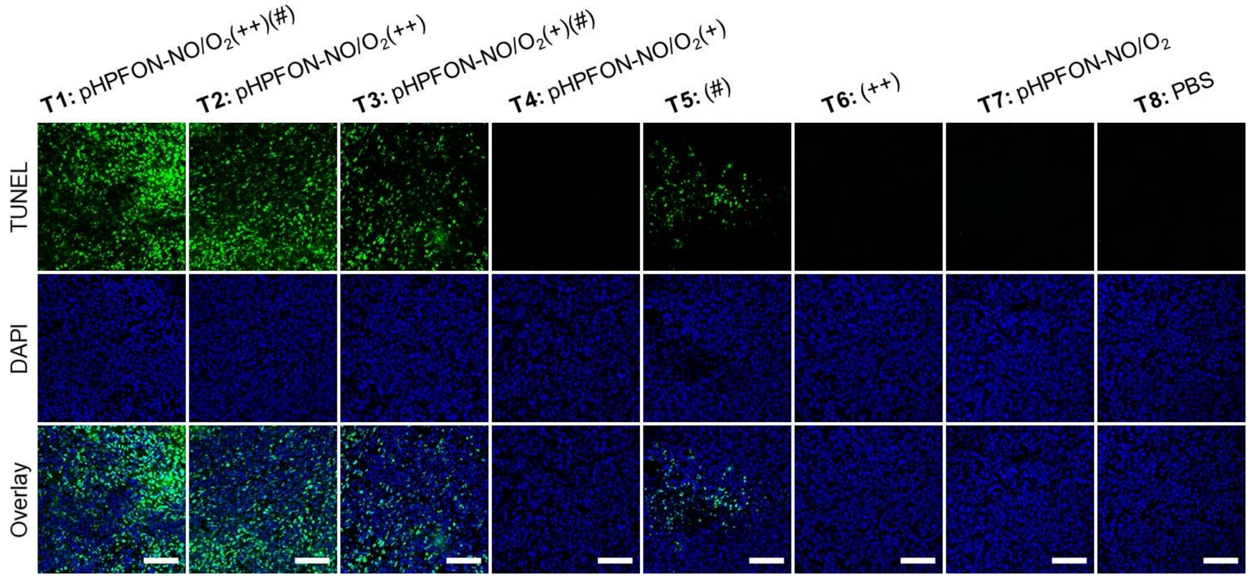

Supplementary Figure 25. TUNEL assays performed on tumor samples acquired at 48 h after different treatments in Fig. 7i. Green, TUNEL. Blue, DAPI. Scale bar, 100 μm. Experiments were performed three times with similar results.

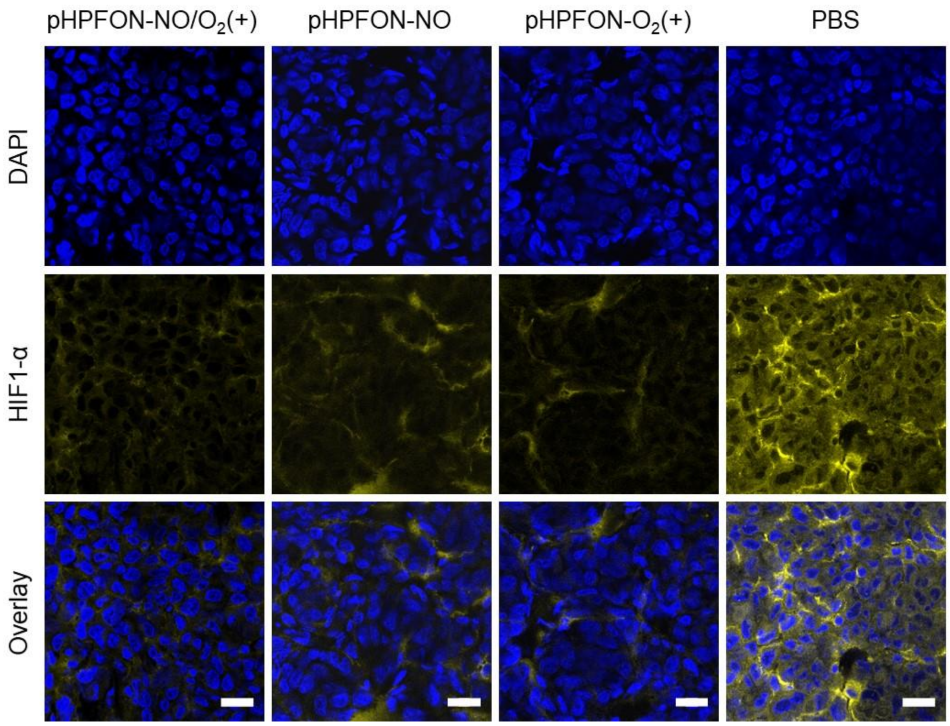

Supplementary Figure 26. Anti-HIF-1α staining on tumor samples acquired at 30 min after different treatments. (+) stands for 808-nm laser irradiation at 1 W/cm^2 for 3 min at 24 h p.i.

Yellow, HIF-1α. Blue, DAPI. Scale bar, 20 μm. Experiments were performed three times with similar results.

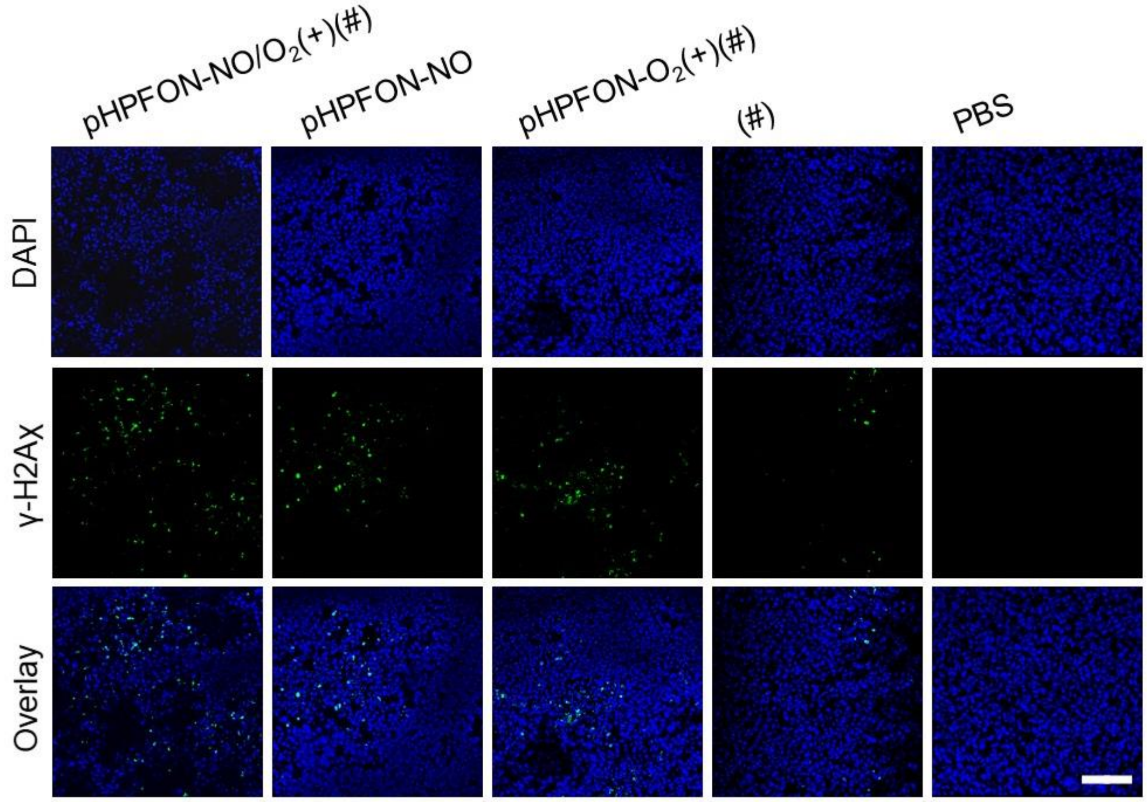

Supplementary Figure 27. Anti-γ-H2Aχ staining on tumor samples acquired at 30 min after different treatments. (+) stands for 808-nm laser irradiation at 1 W/cm^2 for 3 min at 24 h p.i. (#) stands for X-ray irradiation (8 Gy) following the laser irradiation (if applicable). Green, γ-H2Aχ. Blue, DAPI. Scale bar, 100 μm. Experiments were performed three times with similar results.

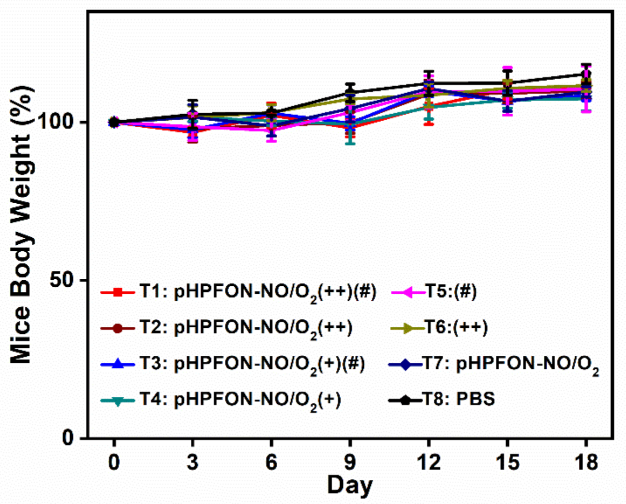

Supplementary Figure 28. Mice body weight changes during the therapy studies. (++) stands for 808-nm laser irradiation at 1 W/cm^2 for 5 min at 24 h p.i. (+) stands for 808-nm laser irradiation at 1 W/cm^2 for 3 min at 24 h p.i. (#) stands for X-ray irradiation (8 Gy) following the laser irradiation (if applicable). n = 5 biologically independent animals. Data are presented as mean ± s.d.

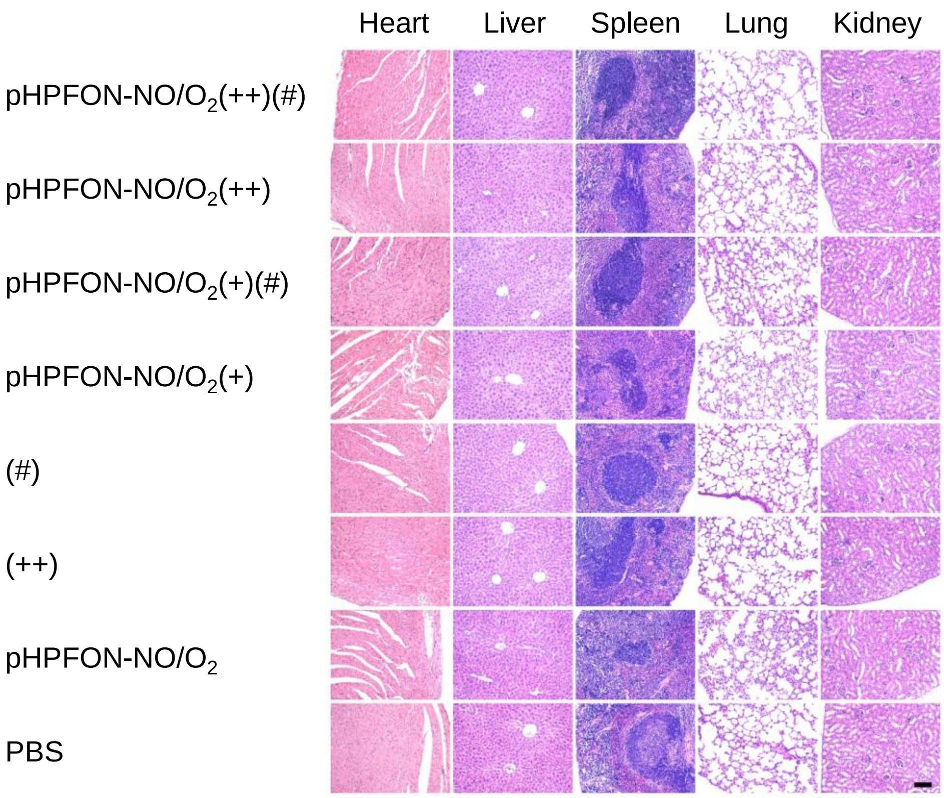

Supplementary Figure 29. H&E staining on major organs (heart, liver, spleen, lung, and kidneys) at the end of the therapy studies. Scale bar, 100 μm. (++) stands for 808-nm laser irradiation at 1 W/cm^2 for 5 min at 24 h p.i. (+) stands for 808-nm laser irradiation at 1 W/cm^2 for 3 min at 24 h p.i. (#) stands for X-ray irradiation (8 Gy) following the laser irradiation (if applicable). Experiments were performed three times with similar results.

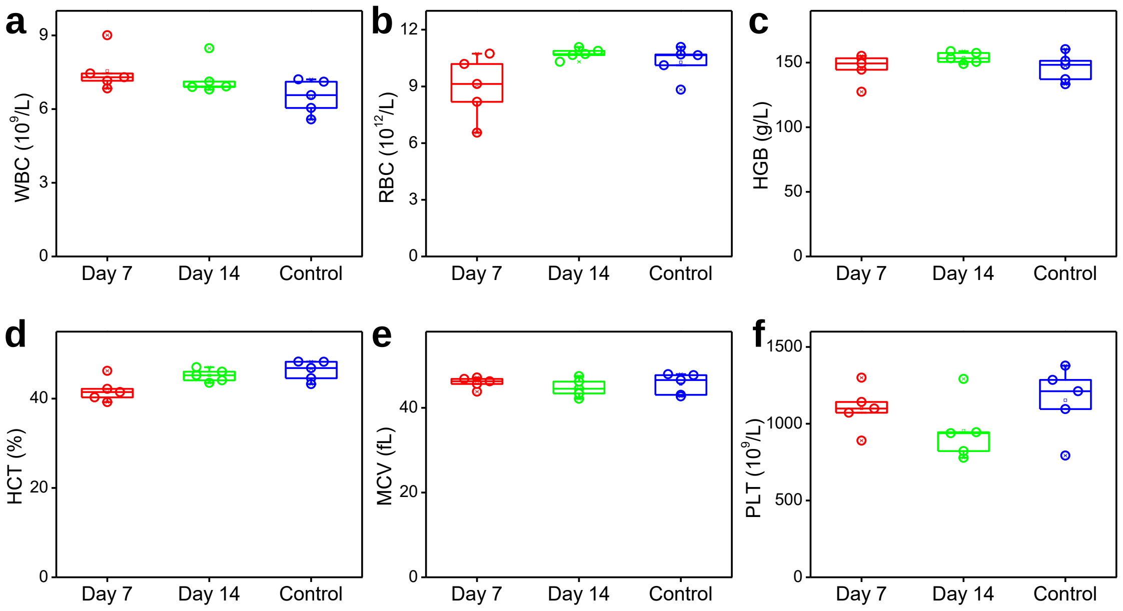

Supplementary Figure 30. Hematological index measurements at day 7 or day 14 p.i. of pHPFON-NO/O_2 in balb/c mice. (a) White blood cells. (b) Red blood cells. (c) Hemoglobin. (d) Hematocrit. (e) Mean corpuscular volume. (f) Platelets. n = 5 biologically independent animals. For the boxplots, the middle line is the median, the lower and upper hinges correspond to the first and third quartiles, and whiskers represent ±1.5 inter-quartile range.

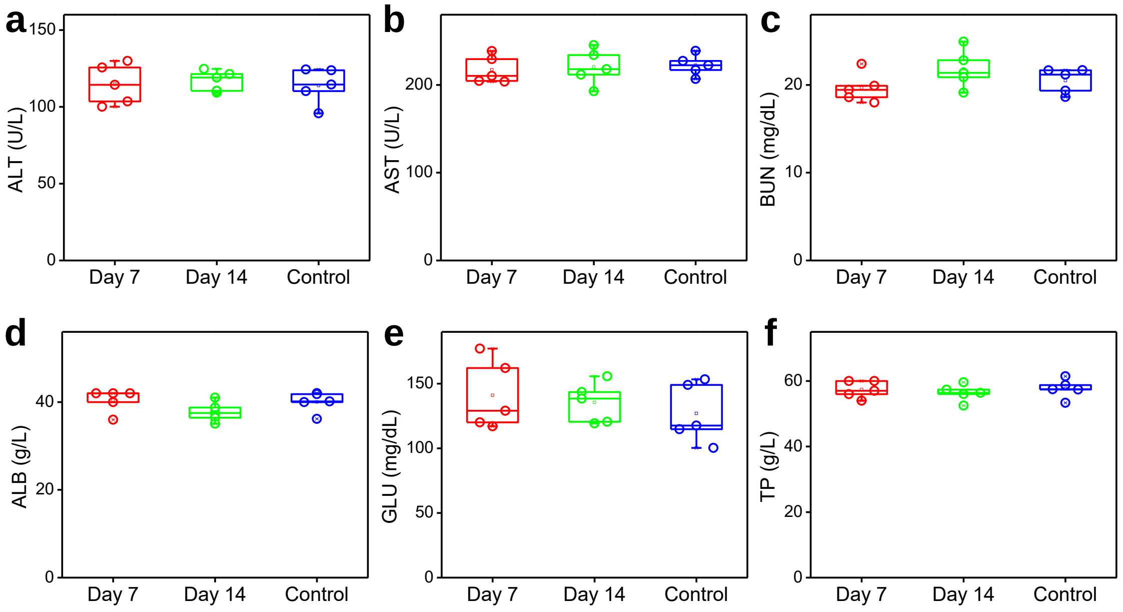

Supplementary Figure 31. Biochemical blood analysis at day 7 or day 14 p.i. of pHPFON-NO/O_2 in balb/c mice. (a) Alanine aminotransferase. (b) Aspartate aminotransferase. (c) Blood urea nitrogen. (d) Albumin. (e) Glucose. (f) Total proteins. n = 5 biologically independent animals. For the boxplots, the middle line is the median, the lower and upper hinges correspond to the first and third quartiles, and whiskers represent ±1.5 inter-quartile range.

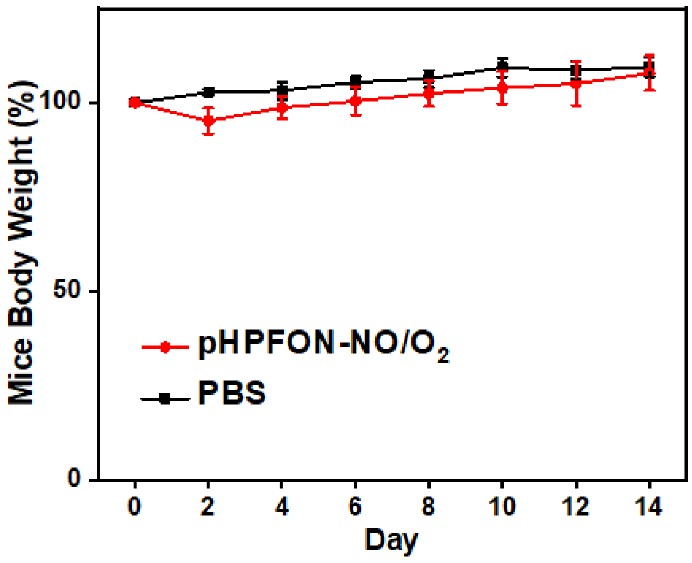

Supplementary Figure 32. Body weight changes of balb/c mice after intravenous injection of pHPFON-NO/O_2 or PBS. n = 5 biologically independent animals. Data are presented as mean ± s.d.

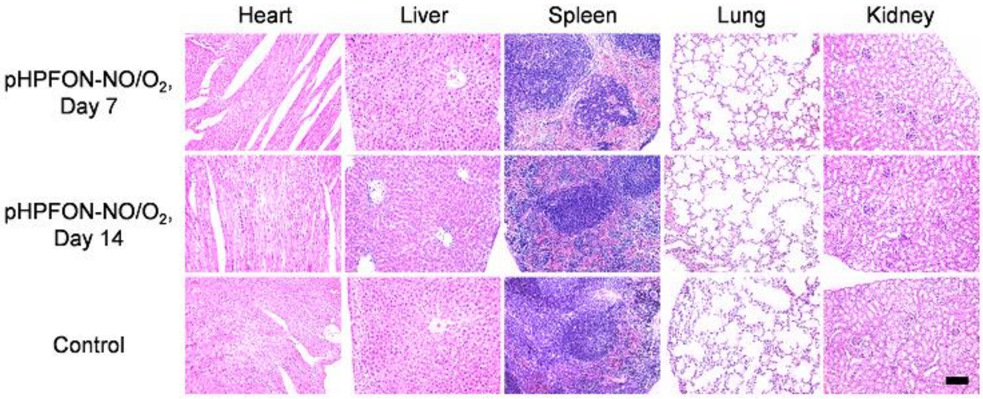

Figure S33. H&E staining on major organs (heart, liver, spleen, lung, and kidneys) of balb/c mice at day 7 or day 14 p.i. Scale bar, 100 µm. Experiments were performed three times with similar results.

Supplementary Methods

Materials. All chemical reagents were purchased from Sigma-Aldrich unless otherwise stated.

2,5-bis(2-ethylhexyl)-3,6-bis(5-(trimethylstannyl)thiophen-2-yl)pyrrolo[3,4-c]pyrrole-1,4(2H,5H)-dione (DPP-Sn) and 2,5-bis(6-bromohexyl)-3,6-bis(5-bromothiophen-2-yl)pyrrolo[3,4-c]pyrrole-1,4(2H,5H)-dione (DPP-Br) were purchased from Derthon Optoelectronic Materials Science Technology Co., LTD. Tris(4,7-diphenyl-1,10-phenanthroline)ruthenium(II) dichloride complex ($[Ru(dpp)_3]Cl_2$) was purchased from Santa Cruz Biotechnology. Alexa Fluor™ 594-conjugated wheat germ agglutinin, DAF-FM Diacetate, JC-1 assay, H_2DCFDA assay, anti-HIF-1α antibody, anti-α tubulin antibody, Alexa Fluor® 488-conjugated goat anti-mouse IgG (H+L) secondary antibody, Alexa Fluor® 594-conjugated goat anti-mouse IgG (H+L) secondary antibody, and LIVE/DEAD™ viability/cytotoxicity kit were purchased from Thermo Fisher Scientific. Anti-γ-$H_2A\chi$ antibody was purchased from Abcam. Griess reagent system was purchased from Promega. FITC Annexin V Apoptosis Detection Kit with PI was purchased from BioLegend. VECTASHIELD antifade mounting medium with DAPI and VECTASHIELD antifade mounting medium were purchased from VECTOR Laboratories. Oxygen consumption rate assay kit and ATP detection assay kit were purchased from Cayman Chemical.

Characterization. Transmission electron microscopy (TEM) images were acquired on a FEI Tecnai T12 electron microscope. Dynamic light scattering (DLS) data was recorded on a Malvern ZetaSizer Nano instrument. UV–Vis absorption spectrum was recorded by using a

Shimadzu UV-2501 spectrophotometer. Fluorescence spectrum was measured on a Hitachi F-7000 fluorescence spectrophotometer. N_2 adsorption−desorption isotherm and corresponding pore size distribution were acquired on a Micrometitics Tristar 3000 system. Photothermal heating images were recorded with a SC300 infrared camera. PA images were acquired with a Visual Sonic Vevo 2100 LAZR system. PET images were performed on a micro Inveon PET scanner (Siemens Healthcare GmbH, Germany). Oxygen concentration in aqueous solution was measured by a MW600 PRO dissolved oxygen meter (Milwaukee Instruments, Inc., NC, USA). Confocal laser scanning microscopy images were obtained on a Zeiss LSM 780 microscopy. Flow cytometry analysis was performed on a BD Accuri C6 flow cytometry.

Cell line and animal models. The U87MG human glioblastoma cell line was obtained from the American Type Culture Collection (ATCC) and cultured with Dulbecco's Modified Eagle Medium supplemented with 1 % Penicillin and streptomycin and 10 % fetal bovine serum. Athymic nude mice and Balb/c mice were purchased from Envigo laboratories. The animals were hosted in equipped animal facility with temperature at 68-79 F and humidity at 30% - 70 %, under the same dark/light cycle (12h:12h). The tumor model was established by subcutaneously injecting around 3×10^6 U87MG cells into the right hind limb of each nude mouse. All the experimental procedures had been conducted by following a protocol approved by the animal care and use committee (ACUC) of the National Institutes of Health Clinical Center (NIHCC).

***In vitro* biocompatibility.** 1×10^4 U87MG cells were seeded in each well of a 96-well plate and incubated at 37 °C overnight. To evaluate the biocompatibility, pHPFON-NO/O_2,

pHPFON-NO, pHPFON-O_2, or pHPFON at different concentrations were added to the plate. After 24 h of incubation, the cells were washed with PBS and subjected to MTT assays. Briefly, the cells were replenished with 100 μL fresh cell culture medium that containing 1 mg/mL of tetrazolium MTT (3-(4, 5-dimethylthiazolyl-2)-2,5-diphenyltetrazolium bromide) reagent. After another 4 h of incubation, the MTT-containing medium was replaced with 150 μL DMSO. The absorbance of each well was measured with a microplate reader at the wavelength of 570 nm.

***In vitro* cellular uptake.** U87MG cells were incubated at 37 °C with FITC-labeled pHPFON-NO/O_2 at a concentration of 200 μg/mL. After different incubation durations (0, 1, 3, or 6 h), the cells were washed with PBS three times. For confocal imaging, the cells were fixed with 4% paraformaldehyde, stained out their membranes with Alexa Fluor™ 594-conjugated wheat germ agglutinin, mounted with mounting medium with DAPI, and examined under confocal microscopy. For flow cytometry analysis, the cells were trypsinized, collected, and finally analyzed.

Anti-HIF-1α staining. The cells or tissues after different treatments were washed with PBS and fixed with 4% paraformaldehyde in PBS for ten minutes. After washing with PBS three times, the samples were permeabilized with 0.1% Triton X-100 in 0.1% sodium citrate aqueous solution for ten minutes. Next, the samples were incubated with 3% bovine serum albumin (BSA) in PBS in a humidified chamber at room temperature for 1 h. Then, the samples were probed with anti-HIF-1α antibody (1 μg/mL) overnight at 4 °C in a humidified chamber. After washing with PBS three times, Alexa Fluor® 488-conjugated goat anti-mouse IgG (H+L) secondary antibody (1 μg/mL) was added to incubate at room temperature for 45

min. For cells, the samples were further incubated with anti-α-tubulin antibody (2 μg/mL) at 37 °C for 1 h and Alexa Fluor® 594-conjugated goat anti-mouse IgG (H+L) secondary antibody (1 μg/mL) at room temperature for 45 min. Cells were then mounted with mounting medium and examined under confocal microscopy. For tissue samples, the slides were mounted with mounting medium with DAPI before the examination.

Anti-γ-$H_2A\chi$ staining. The cells or tumors after different treatments were washed with PBS and fixed with 4% paraformaldehyde in PBS. After washing with PBS three times, the samples were permeabilized with 0.1% Triton X-100 in 0.1% sodium citrate aqueous solution for ten minutes. After blocking with 3% BSA in PBS in a humidified chamber at room temperature for 1 h, the cells were probed with anti-γ-$H_2A\chi$ antibody (2 μg/mL) overnight at 4 °C in a humidified chamber. After washing with PBS three times, the cells were incubated with Alexa Fluor® 488-conjugated goat anti-mouse IgG (H+L) secondary antibody at room temperature for 45 min. Finally, the cells were mounted with mounting medium with DAPI and observed under confocal microscopy.

TUNEL Assay. TUNEL assay for cryogenic sections were performed with an *in situ* cell death detection kit according to the vendor's protocol (Sigma, Catalog #: 11684795910). 15 μm of slides were prepared and fixed with 4% paraformaldehyde in PBS (pH 7.4) for 10 min at room temperature. After washing with PBS, the slides were incubated in permeabilization solution (0.1 % Triton X-100, 0.1 % sodium citrate in water, freshly prepared) on ice for two minutes. After another washing cycle, the tumor samples were incubated with TUNEL reaction mixture in the dark for 60 minutes at 37 °C in a humidified atmosphere. After gently

rising with PBS, the slides were mounted with mounting medium with DAPI and observed under confocal microscopy.

***In vivo* safety evaluation.** 200 μL of 20 mg/mL pHPFON-NO/O_2 was intravenously injected into Balb/c mice (n=5). The mouse body weight was monitored every other day. After 7 or 14 days, mice were sacrificed. Blood was withdrawn for whole blood count and blood biochemical index analysis. Major organs (i.e., heart, kidneys, lung, spleen, and liver) were collected for H&E staining analysis. In the control group, the mice were treated with PBS (n=5).

Statistical Methods. Quantitative data were expressed as mean ± SD. Means were compared using Student's t-test. *$P < 0.05$, **$P < 0.01$, ***$P < 0.001$, and ****$P < 0.0001$ were considered significant, moderately significant, and highly significant, respectively.
