# Supplementary material for: A hybrid semiconducting organosilica-based O2 nanoeconomizer for on-demand synergistic photothermally boosted radiotherapy
Source: Nat Commun. 2021 Jan 22;12:523. doi: 10.1038/s41467-020-20860-3 (PMC7822893; doi:10.1038/s41467-020-20860-3)
Supplement: Supplementary file 3 — Reporting Summary [file 41467_2020_20860_MOESM3_ESM.pdf]

Corresponding author(s): Xiaoyuan Chen, Wenpei Fan, Zhen Yang

Last updated by author(s): Sep 4, 2020

Reporting Summary

Nature Research wishes to improve the reproducibility of the work that we publish. This form provides structure for consistency and transparency in reporting. For further information on Nature Research policies, see our Editorial Policies and the Editorial Policy Checklist.

Statistics

For all statistical analyses, confirm that the following items are present in the figure legend, table legend, main text, or Methods section.

n/a	Confirmed	
☐	☒	The exact sample size (n) for each experimental group/condition, given as a discrete number and unit of measurement
☐	☒	A statement on whether measurements were taken from distinct samples or whether the same sample was measured repeatedly
☐	☒	The statistical test(s) used AND whether they are one- or two-sided<br>*Only common tests should be described solely by name; describe more complex techniques in the Methods section.*
☒	☐	A description of all covariates tested
☒	☐	A description of any assumptions or corrections, such as tests of normality and adjustment for multiple comparisons
☐	☒	A full description of the statistical parameters including central tendency (e.g. means) or other basic estimates (e.g. regression coefficient) AND variation (e.g. standard deviation) or associated estimates of uncertainty (e.g. confidence intervals)
☐	☒	For null hypothesis testing, the test statistic (e.g. F, t, r) with confidence intervals, effect sizes, degrees of freedom and P value noted<br>*Give P values as exact values whenever suitable.*
☒	☐	For Bayesian analysis, information on the choice of priors and Markov chain Monte Carlo settings
☒	☐	For hierarchical and complex designs, identification of the appropriate level for tests and full reporting of outcomes
☒	☐	Estimates of effect sizes (e.g. Cohen's d, Pearson's r), indicating how they were calculated

Our web collection on statistics for biologists contains articles on many of the points above.

Software and code

Policy information about availability of computer code

Data collection	Transmission electron microscopy (TEM) images were acquired on a FEI Tecnai T12 electron microscope. Dynamic light scattering (DLS) data was recorded on a Malvern ZetaSizer Nano instrument. UV–Vis absorption spectrum was recorded by using a Shimadzu UV-2501 spectrophotometer. Fluorescence spectrum was measured on a Hitachi F-7000 fluorescence spectrophotometer. N2 adsorption–desorption isotherm and corresponding pore size distribution were acquired on a Micrometitics Tristar 3000 system. Photothermal heating images were recorded with a SC300 infrared camera. PA images were acquired with a Visual Sonic Vevo 2100 LAZR system. PET images were performed on a micro Inveon PET scanner (Siemens Healthcare GmbH, Germany). Oxygen concentration in aqueous solution was measured by a MW600 PRO dissolved oxygen meter (Milwaukee Instruments, Inc., NC, USA). Confocal laser scanning microscopy images were obtained on a Zeiss LSM 780 microscopy. Flow cytometry analysis was performed on a BD Accuri C6 flow cytometry.
Data analysis	Origin2018/FlowJo_V10/ImageJ (1.43 u, Java 1.6.0_10)/Excel Microsoft 365/Graphpad Prism 7.0/Chembio Draw Ultra 13.0

For manuscripts utilizing custom algorithms or software that are central to the research but not yet described in published literature, software must be made available to editors and reviewers. We strongly encourage code deposition in a community repository (e.g. GitHub). See the Nature Research guidelines for submitting code & software for further information.

Data

Policy information about availability of data

All manuscripts must include a data availability statement. This statement should provide the following information, where applicable:

- Accession codes, unique identifiers, or web links for publicly available datasets
- A list of figures that have associated raw data
- A description of any restrictions on data availability

The authors declare that the main data supporting the findings of this study are available within the article and its Supplementary Information. Extra data are

available from the corresponding authors upon reasonable request.

Field-specific reporting

Please select the one below that is the best fit for your research. If you are not sure, read the appropriate sections before making your selection.

- [x] Life sciences
- [ ] Behavioural & social sciences
- [ ] Ecological, evolutionary & environmental sciences

For a reference copy of the document with all sections, see nature.com/documents/nr-reporting-summary-flat.pdf

Life sciences study design

All studies must disclose on these points even when the disclosure is negative.

Sample size	We calculated the sample size by power analysis.
Data exclusions	No data were excluded from the analysis.
Replication	All attempts at replication were successful. All experiments were performed a minimum of three replicates in independent experiments with similar results, unless further stated.
Randomization	The samples were randomly grouped.
Blinding	The investigators were blinded to group allocation during data collection and analysis.

Reporting for specific materials, systems and methods

We require information from authors about some types of materials, experimental systems and methods used in many studies. Here, indicate whether each material, system or method listed is relevant to your study. If you are not sure if a list item applies to your research, read the appropriate section before selecting a response.

Materials & experimental systems

n/a	Involved in the study	
☐	☒	Antibodies
☐	☒	Eukaryotic cell lines
☒	☐	Palaeontology and archaeology
☐	☒	Animals and other organisms
☒	☐	Human research participants
☒	☐	Clinical data
☒	☐	Dual use research of concern

Methods

n/a	Involved in the study	
☒	☐	ChIP-seq
☐	☒	Flow cytometry
☒	☐	MRI-based neuroimaging

Antibodies

Antibodies used	Anti-HIF-1α antibody (Invitrogen, MA1-516)<br>Anti-α tubulin antibody (Invitrogen, 62204)<br>Alexa Fluor® 488-conjugated goat anti-mouse IgG (H+L) secondary antibody (Invitrogen, A28175)<br>Alexa Fluor® 594-conjugated goat anti-mouse IgG (H+L) secondary antibody (Invitrogen, A-11005)<br>Anti-γ-H2Aχ antibody (Abcam, ab11174)
Validation	All antibodies were verified by the supplier. The quality test data was showed on the manufactures' websites as following.<br>Anti-HIF-1α Antibody (Invitrogen, MA1-516)<br>https://www.thermofisher.com/antibody/product/HIF1A-Antibody-clone-mgc3-Monoclonal/MA1-516<br>Anti-α Tubulin Monoclonal Antibody (Invitrogen, 62204)<br>https://www.thermofisher.com/antibody/product/alpha-Tubulin-Antibody-clone-DM1A-Monoclonal/62204<br>Alexa Fluor 488-conjugated goat anti-Mouse IgG (H+L) Secondary Antibody (Invitrogen, A28175)<br>https://www.thermofisher.com/antibody/product/Goat-anti-Mouse-IgG-H-L-Secondary-Antibody-Recombinant-Polyclonal/A28175<br>Alexa Fluor 594-conjugated goat anti-Mouse IgG (H+L) secondary antibody<br>https://www.thermofisher.com/antibody/product/Goat-anti-Mouse-IgG-H-L-Cross-Adsorbed-Secondary-Antibody-Polyclonal/A-11005<br>Anti-gamma H2A.X antibody (Abcam, ab11174)<br>https://www.abcam.com/gamma-h2ax-phospho-s139-antibody-ab11174.html

Eukaryotic cell lines

Policy information about cell lines

Cell line source(s)	U87MG: ATCC
Authentication	The cells were authenticated by the NIBIB-tissue culture facility for pathogen testing.
Mycoplasma contamination	All the tests were tested to be free of mycoplasma contamination.
Commonly misidentified lines (See ICLAC register)	No commonly misidentified lines were used in the study.

Animals and other organisms

Policy information about studies involving animals; ARRIVE guidelines recommended for reporting animal research

Laboratory animals	Female nude mice, 4-6 weeks old, were purchased from Harlan. The animals were hosted in equipped animal facility with temperature at 68-79 F and humidity at 30%-70%, under the same dark/light cycle (12:12).
Wild animals	This study did not involve wild animals.
Field-collected samples	This study did not involve any samples collected from field.
Ethics oversight	All animal work was conducted in appliance to the NIH Guide for the Care and Use of Animals under protocols approved by the NIH Clinical Center Animal Care and Use Committee (NIHCC/ACUC).

Note that full information on the approval of the study protocol must also be provided in the manuscript.

Flow Cytometry

Plots

Confirm that:

- [x] The axis labels state the marker and fluorochrome used (e.g. CD4-FITC).
- [x] The axis scales are clearly visible. Include numbers along axes only for bottom left plot of group (a 'group' is an analysis of identical markers).
- [x] All plots are contour plots with outliers or pseudocolor plots.
- [x] A numerical value for number of cells or percentage (with statistics) is provided.

Methodology

Sample preparation	The cells after different treatments were washed with PBS, trypsinized, and re-suspended in PBS. The cell suspension was then filtered through a 70-µm cell strainer. The single cell suspension was washed with flow cytometry buffer and then stained with the indicated antibodies.
Instrument	BD Accuri C6 Plus.
Software	FlowJo_V10
Cell population abundance	10000 cells in gate
Gating strategy	based on the values of FSC/SSC

- [x] Tick this box to confirm that a figure exemplifying the gating strategy is provided in the Supplementary Information.
